# Antioxidants in Cocoa

**DOI:** 10.3390/antiox9121230

**Published:** 2020-12-04

**Authors:** Joanna Oracz, Dorota Żyżelewicz

**Affiliations:** Institute of Food Technology and Analysis, Faculty of Biotechnology and Food Sciences, Lodz University of Technology, 4/10 Stefanowskiego Street, 90-924 Lodz, Poland; dorota.zyzelewicz@p.lodz.pl

Cocoa beans are the seeds of the tropical tree *Theobroma cacao* L. Because of the high concentration of bioactive compounds, including antioxidants (polyphenols, tocopherols), they are valued not only in the food industry but also in the pharmaceutical and cosmetic ones [1]. In recent years, interest in these cocoa components has greatly increased because of their potentially beneficial effects on human health. Cocoa antioxidants can inhibit or delay cellular damage either by quenching free radicals or through chelation of transition metal ions, which reduces their capability to form reactive oxygen species. They also exhibit a wide range of physiological properties resulting in protection against diseases, including coronary heart diseases, cancer or neurodegenerative disorders [2,3,4,5]. 

This Special Issue consists of 10 articles related to the effects of genotype and processing conditions on the phenolic compounds profile and antioxidant activity of cocoa derived products, isolation and characterization of antioxidant compounds such as polyphenols and melanoidins from cocoa beans, and assessment of the antioxidant, anti-oxidative stress and anti-inflammatory effects of cocoa beans and cocoa-derived products.

Several studies have well established that processing of cocoa bean including fermentation, drying, alkalization and roasting caused considerable changes in the chemical composition of the final product such as cocoa powder or chocolate [1,6,7,8]. During processing of the cocoa beans, the naturally occurring antioxidants (polyphenols) undergo significant changes in their structure that may impact their bioactivities. Fermentation and drying of the cocoa beans lead to oxidative degradation of polyphenols as a result of contact with the oxidative enzymes, polyphenol oxidase (PPO) and peroxidase. Monomeric flavan-3-ols are enzymatically oxidized to semi-quinones and quinones. Furthermore, these oxidation products are polymerized to condensed high molecular weight insoluble tannins. The native polyphenols may also react with proteins, free amino acids, and mono- or polysaccharides during roasting to form new compounds with antioxidant activities. The major chemical pathways which occur during roasting of cocoa beans and lead to the formation of new molecules are Maillard reactions [1,6,9]. Toro-Uribe et al. [7] focused their research on the understanding the mechanism of inhibition of PPO in cocoa beans, to find the optimal conditions, like concentration of inhibitor, temperature, and time, which enhance inhibition of PPO in cocoa beans without decreasing cocoa polyphenol concentrations. Their results showed that the optimum conditions to obtain the lowest PPO activity and highest total polyphenol content were achieved with 70 mM inhibitory solution (ascorbic acid/L-cysteine) at 96 °C for 6.4 min. Moreover, the described results evidence that heat treatment is a fast and robust method to reduce the activity of PPO enzyme in cocoa beans. As a result, the authors stated that this procedure also increases the extraction yield of polyphenols, and thus can be used to obtain enriched polyphenol extract with low PPO activity. Racine et al. [8] studied the effect of fermentation and roasting on the composition and α-glucosidase inhibitory activity of cocoa beans and powder, and identify the compositional factors and processing conditions that optimize α-glucosidase inhibitory activity of cocoa. They confirmed that processed cocoa powders are promising inhibitors of α-glucosidase, despite a significant reduction in total polyphenol and flavanol concentrations during fermentation and roasting. Due to this, the authors conclude that cocoa processing might generate compounds which enhance cocoa bioactivity, such as Maillard reaction products (MRPs), most notably melanoidins. Fernández-Romero et al. [9] investigated the degradation kinetics parameters of polyphenol and monomeric flavan-3-ols (catechin and epicatechin) during the roasting process of *Criollo* cocoa. The results indicate that degradation kinetics of the total phenolic content and epicatechin showed first-order reactions as the temperature increases, while the catechin showed patterns of formation and degradation. The authors also conclude that roasting at moderate temperatures is necessary to obtain minimal degradation of cocoa polyphenols and consequently antioxidant activities. Urbańska and Kowalska [10] focused their research on the comparison of the polyphenols content and antioxidant activity of chocolates produced from roasted and unroasted cocoa beans from different origins (Ghana, Venezuela, the Dominican Republic, Colombia and Ecuador). The findings demonstrated that the content of polyphenols vary greatly and depends on many factors, both those resulting from the genotype and the geographical and environmental conditions during growth, as well as the technological processes and parameters used. The obtained results indicate that both the beans (roasted and unroasted) and chocolates produced from them exhibited strong free radical-scavenging activity in vitro. In another study, Urbańska et al. [11] examined the effect of conching on the antioxidant potential of chocolate milk masses, taking into account different protein contents in milk obtained by spray or cylindrical drying. The results demonstrate the association between the protein content of milk powder and cocoa mass and the antioxidant potential of chocolate milk masses after conching. The results of these studies show that it is possible to maintain or increase the biological activity of cocoa beans and their derived products (cocoa powder and chocolate) by choosing appropriate processing conditions and cocoa genotype and origin.

Many recent studies revealed that cocoa beans and cocoa-derived products could be considered as an attractive source material for manufacturing of functional foods and nutraceuticals due to their very high content of bioactive compounds, mainly polyphenols, including flavonoids (proanthocyaninidins, monomeric flavan-3-ols, and anthocyanins) and phenolic acids, as well as melanoidins [1,2,3,4,5,7,8,9]. Toro-Uribe et al. [12] focused their research on developing a food-grade and suitable procedure for the recovery of polyphenols from cocoa beans avoiding the degreasing process. The results showed that concentration of ethanol, pH, temperature, irradiation time, and solid-to-solvent ratio affected significantly the yield of methylxanthines, catechins, and procyanidins with a degree of polymerization up to seven, as well as high antioxidant activity determined by oxygen radical absorbance capacity (ORAC). The optimal extraction conditions were 50% (*v/v*) ethanol, pH 6, 70 °C, and 45 min at the solid-to-solvent ratio of 1:120 *w/v*. Thus, they found that ultrasound-assisted solid–liquid extraction is a suitable method for the recovery of cocoa polyphenols and the obtained cocoa extract can be used as a valuable ingredient for functional food, nutraceuticals and cosmetics. Another study that investigated the total phenolic content, antioxidant properties, and structure–activity relationships of high-molecular weight (HMW) melanoidin fractions isolated by dialysis (>12.4 kDa) from raw and roasted under different temperature and relative air humidity conditions, cocoa beans of *Criollo*, *Forastero*, and *Trinitario* beans cultivated in different origins was conducted by Oracz and Żyżelewicz [13]. The results showed that it is possible to enhance the in vitro antioxidant properties of HMW cocoa melanoidins by choosing the appropriate roasting conditions and cocoa type. Moreover, structural analysis confirmed the presence of different bioactive compounds with various mechanisms of action in HMW cocoa melanoidin fractions. These results revealed that the HMW melanoidins fraction from roasted beans of different cocoa types could be considered as a valuable functional ingredient due to its high antioxidant properties in vitro (e.g., reducing power, antioxidant capacity, chelating activity) and total phenolic contents.

Felice et al. [14] paid attention to the antioxidant effect of cocoa husk extract and cherry extract against reactive oxygen species (ROS)-induced oxidative stress in Human Umbilical Vein Endothelial Cells (HUVECs). The results indicate that polyphenols in both extracts are effective in inhibiting ROS. Interestingly, it was also demonstrated that cocoa husk extract possesses an antioxidant effect even at low concentrations. In particular, the authors demonstrated that cocoa husk extract exhibited greater performance on HUVECs and had higher permeability across the rat intestine compared to cherry extract. The results clearly indicate that cocoa husk extract can be utilized as a valuable and cheap source of antioxidants with ROS scavenging ability that may have great relevance in the prevention of cardiovascular diseases (CVD). 

The animal model studies further point to the therapeutic potential of both cocoa extract and cocoa polyphenols in metabolic and cardiovascular alterations. A study reported by Kluknavsky et al. [15] describes the genomic effects of the epicatechin during the stimulation of nitric oxide (NO) release and antioxidant defense in the aorta and left heart ventricle (LHV) investigated by using young male borderline hypertensive rats during two weeks of treatment (100 mg/kg/day p.o.) and two weeks post treatment. The obtained results indicate that a two-week oral administration of epicatechin decreased significantly blood pressure of young male borderline hypertensive rats, and these effects persisted for two weeks after the cessation of the treatment. The authors concluded that the mechanism of the anti-hypertensive effects of epicatechin is considered to be due to the reduced relative content of the iron-containing compounds in the blood, reduced O_2_^•−^ production, and increased nitric oxide synthase (NOS) activity in the aorta and LHV. Finally, Ahmed et al. [16] studied the influence of cocoa flavanols on myocardial injury following acute coronary ischemia-reperfusion. The results demonstrated that 15-day oral administration of cocoa extract containing 250 mg/g flavan-3-ols (procyanidin) to Sprague–Dawley rats protects against myocardial ischemia-reperfusion (I/R) injury and significantly attenuates nitro-oxidative stress, inflammation, and mitigates myocardial apoptosis. It is well established that oxidative stress is considered to reflect an intracellular redox imbalance between the pro- and anti-oxidants, and plays a fundamental role in the pathogenesis of various diseases [15,16]. 

Thus, the findings demonstrated that a diet high in cocoa antioxidants could provide beneficial effects against risk factors of CVD, cancer, and neurodegenerative disorders. In vitro and in vivo studies reported the importance of cocoa antioxidants for the prevention of oxidative stress and inflammation. However, further clinical trials in humans are needed to confirm the health benefits of cocoa antioxidants.
